# Staff training in physical interventions: a literature review

**DOI:** 10.3389/fpsyt.2023.1129039

**Published:** 2023-07-26

**Authors:** Andrew A. McDonnell, Marion C. O’Shea, Stephanie J. Bews-Pugh, Hannah McAulliffe, Roy Deveau

**Affiliations:** ^1^Birmingham City University, Birmingham, United Kingdom; ^2^Studio 3 Clinical Services Limited, Alcester, United Kingdom; ^3^Tizard Centre, University of Kent, Canterbury, United Kingdom

**Keywords:** staff training, physical interventions, restraint, seclusion, seclusion and restraint reduction

## Abstract

**Background:**

Restrictive practices are used frequently by frontline staff in a variety of care contexts, including psychiatric hospitals, children’s services, and support services for older adults and individuals with intellectual and developmental disabilities. Physical restraint has been associated with emotional harm, physical injury to staff and consumers, and has even resulted in death of individuals in care environments. Various interventions have been implemented within care settings with the intention of reducing instances of restraint. One of the most common interventions is staff training that includes some physical intervention skills to support staff to manage crisis situations. Despite physical intervention training being used widely in care services, there is little evidence to support the effectiveness and application of physical interventions. This review will examine the literature regarding outcomes of staff training in physical interventions across care sectors.

**Method:**

A systematic search was conducted following PRISMA guidelines using Cochrane Database, Medline EBSCO, Medline OVID, PsychINFO, and the Web of Science. Main search keywords were staff training, physical intervention, physical restraint. The MMAT was utilised to provide an analytical framework for the included studies.

**Results and discussion:**

Seventeen articles have been included in this literature review. The included studies take place in a range of care settings and comprise a wide range of outcomes and designs. The training programmes examined vary widely in their duration, course content, teaching methods, and extent to which physical skills are taught. Studies were of relatively poor quality. Many descriptions of training programmes did not clearly operationalise the knowledge and skills taught to staff. As such, it is difficult to compare course content across the studies. Few papers described physical interventions in sufficient detail. This review demonstrates that, although staff training is a ‘first response’ to managing health and safety in care settings, there is very little evidence to suggest that staff training in physical intervention skills leads to meaningful outcomes.

## Introduction

### Rationale

The physical restraint, mechanical restraint, and seclusion of individuals in care environments is controversial. Restrictive practices are used frequently by frontline staff in a variety of care settings, including psychiatric hospitals, children’s services, and support services for older adults and individuals with learning disabilities and developmental disorders (1). Restrictive practices can be defined as those that limit freedom of movement, and include involuntary admission, enforced treatment, seclusion, and physical, chemical or mechanical restraint (2). There are ethical and legal issues around the use of restrictive practices that limit human rights, such as freedom of movement and freedom of choice (2). Organisational policies often state that restraints should be reduced and used as the ‘last resort’ (3), while training in physical interventions is frequently presented as a method of reducing the use of restrictive practices, staff training in physical interventions has had limited research emphasis over the years (1, 4).

Manual, mechanical and chemical restraint are in use across care sectors around the world. In a study that looked at acute psychiatric wards in a county in Norway over an eight-year period it was found that restraint was used on 1.7% of admitted patients per year (5). However, rates of restraint can vary quite dramatically. A study looked at nationwide rates of restraint in Pacific Rim countries in which the law requires these are reported and published. Rates of mechanical restraint varied between 0.03 restraint events per million population in New Zealand, 0.17 in Australia, 0.37 in the United States and 98.9 restraint in Japan, representing a variation greater than 3,000 fold (6). A critical review by Fitton and Jones (7) found rates of physical and mechanical restraint varied between 11–78% for periods over 1 year for people with intellectual disabilities living in services (7). In England, National Audit survey data of over 500 National Health Service (NHS) and independent services for people with intellectual disabilities, Sturmey (8) reported that 53% of service-users had been subjected to physical restraints and 10% to seclusion.

Use of restraint and other restrictive practices in schools for children with special educational needs is difficult to determine. A United Kingdom wide survey of families with a child with disabilities carried out by the Challenging Behaviour Foundation (CBF) found that 88% of families reported their child had experienced physical restraint, with 35% reporting that it happened regularly. Seventy-one per cent of the 204 respondents to the CBF survey reported their child had experienced seclusion, 21% of those said this was on a daily basis (9). Services for older adults have shown that incidences of manual and mechanical restraint are not always reported or recorded (10). Issues of definition likely have an impact on reporting. Some restrictive practices are viewed as necessary means to ‘maintain patients’ safety,’ these include the use of bedrails, or leaving a mobility aid out of reach to prevent a patient from ‘wandering.’ While such practices amount to physical restraints they often go unreported (11).

The use of restraint can lead to serious physical harm. In the United States, Weiss (12) reported on 142 deaths in 50 states showing shocking neglect and uncaring use of force, disproportionately impacting young people. Patterson et al. (13) have shown continuing evidence of deaths associated with restraint in the United Kingdom, as well as worldwide. Kersting et al. (14) conducted a systematic review looking at physical harms and death associated with restrictive practices, and found that death, followed by deep vein thrombosis, were the two most common reported physical harms. Emotional harm is also associated with restrictive practices. In a recent literature review, Chieze et al. (2) examined the use of seclusion and restraint in psychiatric services and estimated that incidence of post-traumatic stress disorder (PTSD) following being subjected to these practices is between 25 to 47%. In a systematic review of qualitative studies, Askew et al. (15) note that during seclusion service-users within psychiatric services report feeling vulnerable, neglected, abused, and disconnected, and conclude that seclusion is a risk to mental health. There is an increasing focus on the views of consumers by researchers, and it is becoming increasingly apparent that those on the receiving end of restrictive practices suffer physical and emotional injuries (16–18).

A number of interventions have been implemented in the effort to reduce the use of restrictive practices within care settings. Gaskin et al. (19) undertook a systematic review which included 14 single person case studies. Interventions included staff training, increasing service-users’ preferred activities, and increasing service-users’ choice and control. Restraint and seclusion showed average reductions of 75% in frequency and of 45% in duration, following intervention. Mindfulness training for staff, focusing on self-management and interactional style, has been reported as leading to decreases in use of restraints (20). Post incident review has been reported to decrease use of restraint in some cases, however in other cases it has been reported to increase use of restraint (21). Multi-component models that include a focus on senior leadership, feedback from frontline staff, target setting, outcomes monitoring, and staff training have been shown to reduce the use of restraint (22, 23).

Staff training in crisis management that includes teaching of physical interventions is a common approach in care services (24). Training aims to equip front line staff to safely manage aggressive behaviour, and typically includes both theoretical and practical components, covering topics such as understanding causes of behaviours, recognizing early warning signs, de-escalation techniques and instruction in physical interventions such as physical restraints (25–28). In the United Kingdom, Beech and Leather (29) reviewed the literature regarding workplace violence within healthcare settings and demonstrated that aggression management training is an established health and safety response in most organisations. Whilst training in physical interventions may well be a relatively commonplace response, the evidence for its effectiveness is limited. There have been claims made about a number of variables, including increased confidence (30–34), improved knowledge (30, 35), reduction in staff and patient injuries (36), and reduction in staff illness. While government guidelines and local policies imply that physical interventions are used as a last resort (2), staff training may not always lead to reductions in their use. There is some limited evidence that training in crisis management may even increase the use of physical interventions (37). The development, content, and impact of various training programmes are difficult to explore as many programmes have been modified and renamed over the years (38).

### Objectives and research question

There has been an increasing emphasis in the literature on the reduction of restraint and other restrictive practices (39). In the United Kingdom, the Restraint Reduction Network (RNN) has been attempting to highlight the issues surrounding restrictive practices for people with intellectual disabilities, autism, and other related conditions. If we are to adopt an evidence-based approach to restraint reduction, this will require an understanding of organisational, cultural, and training issues. There have been a limited number of literature reviews that have focused on staff training in physical interventions and its impact (1, 4, 40). The literature is regarded as limited in nature with poorly designed studies (4, 24, 29). Given the importance of reducing restrictive practices, an understanding of the evidence-base for staff training in physical interventions is urgently required. In view of the prevalence of the use of restraint within care settings, its associated harm, and that staff training is an established health and safety response employed within care settings, physical interventions training is a suitable focus for evaluation by systematic review. This review will examine a selected published literature in order to establish the current evidence base for outcomes of staff training in physical interventions across care sectors.

## Methods

### Search methods for identification of studies

Using the Cochrane Database, Medline EBSCO, Medline OVID, PsychINFO, and the Web of Science a systematic literature search was conducted of empirical studies described in English speaking articles published up to January 2021, which examined the effectiveness of staff training in physical interventions within any healthcare service. The search equation was defined using the Boolean connectors “AND” and “OR” following the formulation “staff training” AND “physical intervention” OR (“physical restraint,” “aggression,” “violence” AND “learning disability,” “intellectual disability,” “developmental disability,” “mental handicap,” “elderly,” “education,” “psychiatry,” “mental health,” “disruptive behaviour”). To broaden our search, websites of 17 training organisations that deliver training services for people with a learning disability in the United Kingdom were examined for evidence of relevant research in staff training in physical interventions. Furthermore, we performed a manual search of the reference list of all studies selected for the review.

### Inclusion and exclusion criteria

Articles were selected for inclusion in the review if they met the following criteria: (i) they were published in a peer reviewed journal; (ii) there was evidence that staff training had taken place and included training in physical interventions (iii) the study utilised a control or a comparison group.

### Final studies included

**Figure 1:**
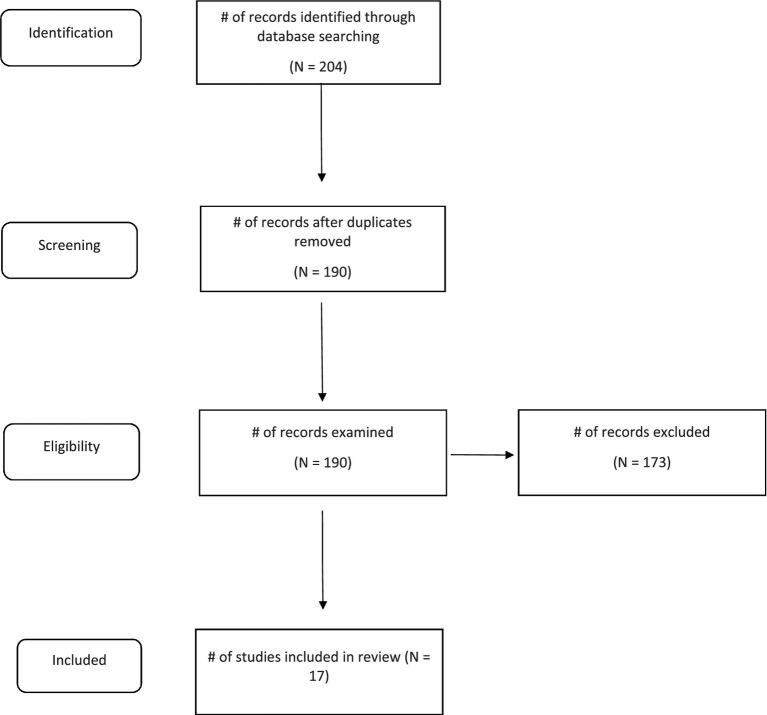
PRISMA flow diagram.

Due to the heterogeneity of the studies, a formal quantitative synthesis (i.e., meta-analysis) was not possible. Instead, a systematic review was conducted using the Preferred Reporting for Systematic Reviews and Meta-Analyses (PRISMA) guidelines (41). The initial search identified 204 papers. 190 papers remained after duplicates were removed. These papers were examined against the inclusion and exclusion criteria, resulting in 173 papers being excluded. 17 papers remained and are included in the literature review. A clear description of the process can be seen in the PRISMA flow diagram (Figure 1). Included studies were further checked for methodological quality by using the Mixed Methods Appraisal Tool (MMAT) and an overall quality score between 1 (indicating relatively poor quality) and 5 (indicating relatively high quality) was assigned to each study (42).

## Results

Seventeen studies met inclusion criteria and are included in the literature review. Table 1 presents titles of training courses, setting, course content, physical interventions taught, and teaching methods described across the studies. A summary of study designs, measures utilised, and outcomes reported can be seen in Table 2.

**Table 1:** Course content, physical interventions taught, and teaching methods described.

**Author**	**Title of training course, duration and setting**	**Course Content**	**Description of Physical Interventions**	**Description of Teaching Methods**
Allen & Tynan (30)	*Preventing and Responding to Aggressive Behaviour* (also referred to as *The Welsh Method);* 2-3 days; Community services for people with learning disabilities, UK.	1 day theory, 2-3 days physical skills; Preventative strategies with a focus on environmental actors and antecedent events; Reactive strategies, gradient of responses from less intrusive (distraction, diffusion, relaxation) to more intrusive (self -protective procedures and minimal restraint); Minimalist approach - staff only taught defensive/restrictive holds relevant to individuals hey support.	Not specified	Didactic, videos of role plays, group work, individual assignments; Physical skills were taught in a in a gym setting.
Carmel & Hunter (43)	*Management of Assaultive Behaviour,* The California Department of Mental Health Training; 16 hours; 973 bed forensic hospital, USA.	Included teaching of “interpersonal skills,” and “instruction in the management of violent patients.”	Not specified	Didactic, lecture-based and practical instruction.
Chang et al. (44)	Untitled course given as part of a multicomponent intervention programme, described as being developed from evidence-based literature; 2 hours; 4 Intensive Care Units (ICUs), Taiwan.	Types of physical restraints, how to choose physical restraint method restraint and precautions, instructions and guidelines, alternative approaches and ethical and legal issues.	Not specified	Narration, question-and-answer, technical demonstration, and open discussion.
Craig & Sanders (45)	*The Grafton Model,d*escribed as a multi-component approach, which includes a focus on staff communication, support and debriefing in addition to training; Duration of training course is not stated, but training is described as being given ‘in-situ,’ during and after incidents; Evaluation of Grafton’s services, including psychiatric residential and community group homes, education, outpatient and early intervention services provided 3244 to children and adults, USA.	Teaching knowledge and skills needed to practically implement a philosophy of ‘comfort-verses-control,’ minimizing the use of restraint and seclusion, and keeping clients and employees safe; Focus on trauma informed approaches, facilitation of growth and rehabilitation.	Training in blocking techniques that are described as ‘organizationally created,’ including the use of pillows, cushions, bean bags and other soft objects; Use of blocking techniques as an alternative to physical restraint and seclusion.	Didactic teaching; Increased support staff allowed for modelling and coaching when crisis situations occurred; Debriefing after incidences of restraint and seclusion focused on antecedents to avoid and supports needed to prevent future incidents.
Godfrey et al. (46)	*Nonviolent Crisis Intervention* from the Crisis Prevention Institute (CPI); 16 hours; 398 bed state psychiatric hospital, USA.	De-escalation techniques, prevention and management of aggressive behaviour; Identification of early signs of escalating behaviour; Strategies for avoiding power struggles and for setting limits; Physical interventions taught to be used ‘as last resort.’	Practiced self-defence manoeuvrers	Practiced models to use when confronted with anxious, hostile, or violent behaviour.
Hahn, Needham, Abserhalden et al (47)	*Aggression management training* *programme*; 5 days / 24 sessions; 3 Acute Psychiatric Hospitals, Switzerland.	Definitions, nature, prevalence, and theories of aggression; Nursing care plans; Nursing interventions, including prediction and prevention, communication, breakaway techniques, use of measures to restrict patient’s freedom; Post incident care, ethics of aggression management, ward security.	Breakaway techniques	Problem based learning; Theoretical elements and exchange of experience; Hands on training.
Hurlebaus & Link (48)	*Managing Aggressive Behaviour;* 4 hours Nurse training course, USA.	Crime and motivation to crime (as relevant to institution); Definitions of anger and theories of aggression; Verbal and non-verbal signs, antecedents, verbal and non-verbal communication skills; Non-physical interventions and prevention emphasised as preferred means; Brief overview of legalities.	Self-defence skills adopted from various martial arts disciplines; Breakaway techniques, including from wrist grabs, chokes (front and rear) and hair pulling, blocking kicks, ‘how to release from a bite’; Restraint.	Didactic, information pack, discussion and discussion; Demonstration of verbal and non-verbal communication skills; Demonstration and supervised practice of physical skills.
Infantino & Musingo (49)	*Aggression Control Techniques (ACT);* 3 days; Psychiatric hospital, USA.	Local policy and procedures related to patient rights; Verbal de-escalation strategies; Instructions in basic physical interventions, restraint, control, transport of patients, and incident reporting procedures.	Basic physical intervention techniques designed to provide staff with effective release and escape skills, such as from hair pulling, choking, and headlocks, and blocking punches and kicks; Restraint and control techniques not described.	Methods to teach verbal interventions include case vignettes and role-play; Videos used to demonstrate skills, including verbal de-escalation, escape, and restraint; Other teaching aids include a guide for the instructor and a student text, both incorporate learning and performance objectives and scoring criteria, which are used to assess staff’s competency after completion of the training.
McDonnell, Sturmey, Oliver, et al (34)	*Managing Challenging Behaviour (MCB)* from Studio III Training Systems; 3 days; 2 residential social care and day services for people with Autism Spectrum Disorders (ASD), UK.	Focus on preventative strategies and Low-Arousal approach to de-escalation and reduction of aggressive behaviour; Legal and ethical issues, causes of aggressive behaviour, staff support; Responding to high frequency aggressive behaviour, e.g. hair pulling, biting, grabbing.	Physical interventions designed to reduce pain and to pass the ‘social validity’ test; e.g. airway protection, two person seated chair restraint in an upright posture.	Lectures; Modelling strategies with rehearsal using role play.
McGowan, Wynaden, Harding et al (50)	*Safe physical restraint;* 7.5 hours; Psychiatric intensive care units, Australia.	Early recognition and management of antecedent behaviours, diffusion techniques, empowerment of patients to take control of their behaviour, teamwork and role assignment during the restraint process, guidelines and standards for safety of patients and staff.	Not specified	Lecture based methods implied but not clearly specified; Role play scenarios.
Needham, Abderhalden, Zeller, et al. (51)	*Management of aggression*; Training consisted of 20 x 50 minute sessions over 4 days; Health care settings, Switzerland.	Definitions, nature, prevalence, and theories of aggression; Staff reflection on own aggressive components; Theory on stages of aggressive incidents, behaviour during aggressive situations, types of conflict management, communication, post aggression procedures. workplace safety.	Breakaway techniques; Physical skills not specified.	Lecture based and role play
Needham, Abderhalden, Halfens et al. (52)	*Management of aggression* (Oud, 1997); Training consisted of 20 x 50 minute sessions over 4 days; 6 Acute Psychiatric Wards in the German speaking regions of Switzerland.	Definitions, nature, prevalence, and theories of aggression; Staff reflection on own aggressive components; Theory on stages of aggressive incidents, behaviour during aggressive situations, types of conflict management, communication, post aggression procedures. workplace safety.	Breakaway techniques; Physical skills not specified.	Lecture based and role play: Train the trainer model.
Phillips & Rudestam (53)	Untitled training programme; 4 hours 20 minutes; 2 state psychiatric hospitals, USA.	Theories of learning, dynamics of violence, warning signs of violence, non-verbal communication, intervention strategies and legal issues	The development of increased skill in nonviolent physical techniques of self-defence; There are clear descriptions of 2 physical interventions, designed to ‘repel’ and escape from physical aggression.	Lecture and role play
Rice, Helzel, Varney et al (54)	*Crisis Prevention and Intervention;* 5 days; Psychiatric hospital, Canada.	Prevention: situations likely to trigger agitation, recognising behavioural cues indicative of agitation; Verbal techniques to diffuse aggression; Self-defence techniques; Safe and effective methods for restraint; Post incident mediation and learning.	Self-defence techniques and methods for physical restraint not specified.	Lecture based; Videotaped simulations of effective and ineffective methods of dealing with crisis situations; Role play.
Testad, Aasland & Aarsland (55)	Untitled manualised training programme; 6 hour seminar; Additional guidance was then provided for one hour every month, for 7 months; 4 nursing homes for people with dementia, Norway.	Seminar focused on dementia, aggression, problem behaviour, decision making process, and alternatives to the use of restraint; Data and information were presented for each individual patient, who were then considered based on the topics in the seminar and individualised care plans developed.	Not clearly specified.	lecture based, guidance, discussion.
Thackrey (56)	*Therapeutics for Aggression;* 8 hours, consisting of 2 x 4 hour sessions one week apart; A Community mental health centre, a state psychiatric prison, and a state psychiatric hospital, USA.	Legal and ethical issues; Principles of psychological assessment and intervention; Teamwork and communication skills; Physical methods for non-abusive self-protection and patient control.	Not specified.	Didactic lecture, selected readings, group discussion, experiential exercise, role play, practice of physical protection and control manoeuvres.
Van Den Pol, Reed & Fuqua (57)	*Emergency Procedures;* 1 hour 30 minutes – 3 hours, consisting of 3 x 30-60 minute workshops; 87 bed residential service for people with a learning disabilities, USA.	Emergency procedures for responding to a facility fire, patent seizure and patient physical aggression were taught in their component steps; Assessment includes trainee having to demonstrate essential skills.	Clear descriptions of physical interventions; e.g. blocking punches, blocking kicks, releasing a clothing grab using a “thumb pry,” release of a body part grab; using a chair for protection	Train the trainer model, didactic lecture, handout, working 1:1 until mastery in emergency procedures demonstrated.

**Table 2:** Study design, measures utilised, and outcomes reported.

**Author**	**Design**	**Measures**	**Outcomes reported**	**Overall quality score assigned**
Allen & Tynan (30)	Quasi-experimental design, pre-test post-test; Participants were 109 staff working in Community services for people with learning disabilities; Experimental group (n=51) received training; Control group (n=58) did not receive training; Non-parametric statistics used.	*Confidence in Coping with Patient Aggression Instrument (CCPAI)*; *Reactive Strategy Questionnaire*, designed for this study to assess knowledge of reactive behaviour management.	Trained group was significantly more confident than untrained group; Trained group scored higher on reactive strategy questionnaire; Both measures statistically increased when untrained group received training.	5
Carmel & Hunter (43)	Quasi-experimental design; Participants were 744 staff working in a forensic hospital; Experimental group (n=392) compliant with training requirements in managing assaultive behaviour; Control group (n=352) staff who were non-compliant with training requirements in managing assaultive behaviour; Also compared low and high compliance wards; Parametric statistics used.	Staff injury data; Rates of patient aggression.	Staff compliant with training requirements in the Management of Assaultive Behaviour were less likely to be injured than staff who were non-compliant (11% compared to 18.2% injured (p<.005 Fisher's exact test) No significant difference between the low-compliance wards and the high-compliance wards in the number of aggressive incidents per bed Rate of staff injury from patient violence in the wards with low compliance with training in managing assaultive behavior (20.0 per 100 staff) was almost three times the rate in the wards with high compliance (7.4 per 100 staff) (t = 2.77, df= 24, p<.005).	4
Chang et al. (44)	Quasi-experimental design, pre-test post-test; Participants were 136 nursing staff from 4 Intensive Care Units (ICUs), Taiwan; Experimental Group (n=76) received training; Control Group (n=60) did not receive training; Semi-parametric tests used (General Estimation Equation).	10 item Questionnaire for knowledge about physical restraints; Questionnaire for attitudes about physical restraints); Questionnaire for behaviour related to physical restraints, modified from Restraint Behaviour Questionnaire; An observation tool for technical skill for physical restraints carried out correctly to set protocol.	In the experimental group, post test scores for knowledge and technique were significantly higher than the pre test scores In the experimental group, post test scores for attitudes and behaviors did not significantly differ from pre test scores No changes found in the control group	5
Craig & Sanders (45)	Longitudinal service evaluation of restraint reduction program; Included in the evaluation were the Grafton services that had more than 750 employees, these services pertained to 3244 service users in 2016; Context, Input, Process, Product (CIPP) evaluation model with a data validation design to analyse documents, quantitative data, and qualitative data from interviews.	Audit data included restraint frequency, client induced injuries, staff injuries from restraints, lost time and modified duty, lost time expense, annual workers compensation costs, cost of employee turnover, total return on investment data, restraint and seclusion incidents, client goal mastery (average of client goal attainment compared to the number of client goals closed throughout the fiscal year).	99% decrease in restraints used from 2003 through 2016, including a 97% decrease of restraint in community-based programs and 90% decrease in residential treatment centres. A newly acquired treatment facility (2011) accompanied an increase and subsequent decrease in restraints over the reported period. 100% decrease in seclusion 2003 -2016 (fell from 253 to 0) in residential treatment facilities. 97% decrease in staff injuries due to restraint (110 to 3) from 2004 to 2016 across all services.	5
Godfrey et al. (46)	Longitudinal evaluation of restraint reduction program over 3 years; Included in the evaluation were patients (n=2910) admitted to an Acute Adult Unit (AAU) and patients (n=334) admitted to a Community Transition Unit (CTU) September 2009-July 2012; Phase 1: All staff received 16 hours training, and to ‘ensure fidelity of the model’ there was use of a specialised response team consisting of staff with advanced training to assist in crisis situations; Phase 2: Policy change requiring management approval for use of mechanical restraint; Parametric tests used.	Daily incidence rates were collated for use of mechanical restraint (MR), seclusion, manual holds, PRN medications administered and assaults to patients, staff and property; Number of injuries to staff and patients as a result of assault or containment were also examined; No reliability data reported.	Following phase 1, mechanical restraint was significantly reduced in both AAU and CTU services. Following phase 2, mechanical restraint was again significantly reduced in AAU, but not in CTU as it’s use had already eliminated. Overall results are reported: Mechanical restraint reduced by 100% in CTU and 98% in AAU AAU showed a decrease in rates of seclusion and use of PRN medication CTU showed an increase in rates of seclusion and use of PRN medication CTU showed an increase in numbers of injuries to staff and consumers. AAU & CTU showed no significant change in use of manual holds	5
Hahn, Needham, Abserhalden et al (47)	Quasi-experimental design, pre-test post-test; Participants were 63 mental health nurses in an Acute Psychiatric Hospital, Switzerland; Experimental group (n=29) were staff working on three wards in 2 hospitals and received training; Control group (n=34) were staff working in 3 wards in 1 hospital did not receive training; Non-parametric tests used.	Management of Aggression and Violence Attitude Scale (MAVAS).	No significant changes were found in attitudes to the management of aggression and violence	4
Hurlebaus & Link (48)	Quasi-experimental design, pre-test post-test; Participants were trainee nurses (n=32); Experimental group (n=22) received training in managing aggressive behaviour; Control group (n=10) did not receive training; Parametric statistics used.	15-item knowledge test (consisting of 10 multiple choice and 5 true/false questions); Visual analogue scale to measure safety; Visual analogue scale to measure confidence.	Significant difference in measures of knowledge pre and post training in the experimental group No significant differences found in measures of safety or confidence	3
Infantino & Musingo (49)	Quasi-experimental design, pre-test post-test; Participants were unit staff and supervisors (n=96); Experimental group received training (n=31); Control group (n=65); Followed up 9 months to 2 years after training; Non-parametric statistics used.	Examined rates of staff assaults, injuries and days lost from work.	Reported that 1 trained member of staff was assaulted with no injury 37% of the untrained staff were assaulted, 79% of these resulted in injuries. Staff that participated in training were significantly less likely to be assaulted An association is reported between participating in training and not being injured, but this was not statistically significant	3
McDonnell, Sturmey, Oliver, et al (34)	Quasi-experimental design, pre-test post-test; Participants were staff from 2 services (n=90); Experimental group (n=43) received training; Control group (n=47) had received training prior to the study period; Parametric tests used.	*Staff Support and Satisfaction Questionnaire (3SQ)*; *Shortened Ways of Coping (Revised) Questionnaire (SWC-R)*; *Thoughts about challenging behaviour questionnaire*; *Challenging behaviour confidence scale*; *Checklist of challenging behaviour*.	Staff training showed increases in staff confidence but not other measures of staff belief, support, coping or perceived control. No evidence of reduction in client challenging behaviour as staff in both groups reported reductions in challenging behaviour	4
McGowan, Wynaden, Harding et al (50)	Benchmarking study; Participants were staff from 3 intensive psychiatric care units at 2 hospitals (n=70); Experimental group (n=28) received training; Control group (n=42) were already in receipt of regular training; Non-parametric statistics used.	*Confidence in Coping with Patient Aggression Instrument (CCPAI)*.	The control group who were in receipt of regular training had higher confidence scores, than the untrained group. When the untrained group later received the intervention, their confidence scores significantly increased after training.	1
Needham, Abderhalden, Zeller, et al. (51)	Quasi-experimental design, pre-test post-test; Participants were student nurses (n=117); Experimental group (n=57) received training; Control group (n=60) did not receive training; Parametric and non-parametric statistics used.	*Confidence in Coping with Patient Aggression Instrument (CCPAI)*. *Perception of Aggression Scale Shortened (POAS-S*).	Experimental group – demonstrated enhanced confidence but no change in attitude after the training course	4
Needham, Abderhalden, Halfens et al. (52)	Cluster randomised control trial; Participants were nurses from 6 Acute Psychiatric Wards (n=68); Experimental group (n=30) received training; Control group (n=28) did not receive training until after completion of the study; Parametric and non-parametric statistics used.	*Perception of Aggression Scale Shortened (POAS-S*); *Tolerance Scale*; *Impact of Patient Aggression on Carer Scale* (IMPACS).	No differences were found between groups, and no differences were found pre and post training in the intervention group.	3
Phillips & Rudestam (53)	Quasi-experimental design, pre-test post-test; Participants were male staff at 2 two psychiatric hospitals (n=24); Experimental group 1 received didactic training plus training in physical (n = 8); Experimental group 2 received didactic training (n = 8); Control group did not receive training (n=8); Parametric statistics used.	*Buss-Durkee Hostility-Guilt Inventory*; Questionnaire about clinical experience and sports training; Judges' evaluations of physical skill, aggression, and fear (high inter-rater reliability reported); Self-report questionnaire about the value of nonaggressive responses, felt fear and aggression; Follow-up questionnaire about post training assaults.	Judges ratings of behaviourally expressed fear and aggression were significantly reduced in experimental group 1 (received both didactic and physical skills training) No significant changes were found in Judges ratings of behaviourally expressed fear and aggression in experimental group 2 (didactic training) or in the control group Follow-up interviews indicated that staff in experimental group 1 (received both didactic and physical skills training) reported 23% fewer incidents of assault compared to staff who received only didactic training, and 20% fewer incidents of assault than staff who received no training	3
Rice, Helzel, Varney et al (54)	Quasi-experimental design, pre-test post-test; Participants were staff (mostly registered nursing assistants) and volunteers at a psychiatric hospital (n=125); Experimental group (n=88) were male staff (n=62) from 4 maximum security wards and male and female staff (n=26) from two wards of the lesser security division of the hospital; Control group (n=37) were male volunteers (n=14) from another maximum security unit of the hospital and male and female volunteers (n=23) from the lesser security division of the hospital; Parametric and non-parametric statistics used.	Staff/volunteer measures designed specifically to evaluate this training course in these settings: Sensitive situations skill test; Audiotaped simulations test; Physical skill test; Self defence and patient restraint written test; Course feedback questionnaire; On-Ward reactions scale; Patient measures: *Coopersmith Self Esteem Inventory*; Modified version of the *Feelings Scale*; Data collated for: Assault rates; Assault rates leading to days off work.	Increases in performance in all pre-post simulations and written tests Increases in ratings on the On-Ward reactions scale for staff from the maximum security wards Significant reduction in physical assaults / incidents Significant reduction in workdays lost due to patient violence Course feedback is reported as positive, and remaining positive at 15-month follow-up.	3
Testad, Aasland & Aarsland (55)	Cluster randomised control trial; Participants in this study were residents in 4 residential and nursing homes for people with dementia; Experimental group (n=55) residents in 2 homes who’s caregivers received training; Control group (n=96) residents in 2 homes who’s caregivers did not receive training; Non-parametric statistics used.	Brief Agitation Rating Scale (BARS); Frequency of use of restraint assessed by a standardised interview.	At baseline the number of restraints and BARS scores did not differ At follow up the use of restraint was significantly lower in the intervention group compared to the control group, reducing the number of incidents of use of restraint by 54% BARS scores significantly increased in the intervention group	3
Thackrey (56)	Quasi-experimental, pre-test post-test design with an 18 month follow up; Participants were staff (n=125) at a community mental health centre, a state psychiatric prison, and a state psychiatric hospital; Experimental group (n=68) received training; Control group (n=57) did not receive training; Parametric statistics used.	*Confidence in coping with patient’s aggression instrument*.	The trained group reported a gain in confidence that was maintained at the 18-month follow-up The untrained group showed no significant changes under the three time periods	4
Van Den Pol, Reed & Fuqua (57)	Quasi-experimental, pre-test post-test design with a maintenance condition; Participants were staff (n=13); Experimental group (n=8), consisted of staff (n=4) who had been in post >6 months (trainers) who were trained to train newer staff (n=4) (trainees); Control group (n=2) new staff who did not receive training (control trainees); Maintenance condition (n=3) new staff trained by trainees, who were trained previously in the experimental condition; Descriptive statistics.	Skill acquisition: Role-play assessments of self-defence procedures rated by 2 independent raters (inter-rater reliability reported as 90%); Assessments took place on an unannounced basis; 5-item self-report questionnaire to assess Acceptability; Social validity assessed via telephone follow-up of staff still employed at the facility 23 months later (number of staff not specified).	Pre-training assessment of trainees (n=4) relating to correct steps followed for physical skills is reported as 43%; Post-training assessment of trainees (n=4) relating to correct steps followed for physical skills is reported as near to 100%; Control trainees showed no increase in any skill acquisition; In the maintenance condition it is reported that mastery is maintained for trainees who go on to train peers in physical skills (n=1); Mastery was also maintained by another trainee who did not go on to train their peers in physical skills (n=1).	4

### Descriptions of settings and client-groups

Studies took place in a variety of settings and with different population groups (see Table 1). Ten studies were carried out in adult psychiatric settings (43, 46, 47, 49–54, 56); three in adult learning disabilities settings (30, 45, 57); one in a service for older adults (55); and one in a service for adults with autism (34). Two studies took place in general hospitals (44, 48), one of which was an Intensive Care Unit (ICU) (44). Craig and Sanders (45) examined multiple services provided by one organisation for children and adults with intellectual and developmental disabilities and psychiatric needs. Studies were conducted in a variety of locations, including the United States (43, 45, 46, 48, 49, 56, 57); Switzerland (47, 51, 52); United Kingdom (30, 34); Canada (53, 54); Australia (50); Taiwan (44); and Norway (55). The number of participants in each study varied widely from thirteen (57) to 1,488 (43).

### Training systems utilized

The majority of physical intervention training is provided under a brand name by commercial organisations (see Table 1). Two studies (46, 54), reported using a training course from the Crisis Prevention Institute (CPI). Three studies (47, 51, 52), reported using the *Aggression Management Training Programme* (Oud, 1997). One study reported using each of the following branded training: Studio III training (34); *The Welsh Method* (30); *The Grafton Method* (45) *Aggression Control Techniques (ACT)* (49); *Management of Assaultive Behaviour* (43); *Safe Physical Restraint* (50); *Therapeutics for Aggression* (56); and *Emergency Procedures* (57). Four studies did not describe a recognised training programme (44, 48, 53, 55).

The duration of the training courses ranged from less than 1 day to more than 5 days. Five studies reported training courses that were less than 1 day (44, 45, 48, 53, 57); three reported providing 1 day workshops (50, 55, 56); two reported 2 day courses, (43, 46); two reported 3 day courses (34, 49); one study specified two to 3 days training depending on need (30); and four reported five or more day courses (47, 51, 52, 54).

### Teaching methods

A variety of teaching methods were described (see Table 1). Twelve studies reported using lecture or classroom-based formats (30, 34, 43, 44, 46–48, 51, 53–56). Four studies reported utilising groupwork or discussions, such as question and answer sessions (30, 44, 48, 56). Three studies reported use of audio-visual aids (30, 49, 54). Ten studies reported using role-play (30, 34, 49, 50–54, 56, 57). One study referred to ‘hands on training,’ but no further details were provided (47). One study reported using behavioural skills training (57). Two studies made reference to the use of additional teaching materials, such as manuals, incident books and theoretical information (48, 49). Two studies referred to physical demonstrations of physical restraint skills (44, 46). Two studies did not clearly outline teaching methods (45, 46).

### Course content

Diffusion strategies were reported to have been taught on 14 training courses (30, 34, 43–46, 48–56). Four of these studies reported training in communication skills, including verbal and nonverbal, and psychological de-escalation techniques (45, 46, 48, 52). Another study discussed that training included a consideration of precautions when choosing a restraint method, consideration of alternative approaches, and ethical and legal issues (44). One study described training that included imparting knowledge and skills needed to practically implement a philosophy of comfort versus control (45).

In general, the included studies did not provide clear descriptions of physical interventions, with only three studies clearly describing the physical techniques taught (34, 53, 57). Nine studies did not provide a list of the specific physical interventions taught on training courses (43, 46, 47, 50, 51, 54–56), and five studies referred to other source materials to describe their physical interventions (30, 34, 45, 51, 52).

Four studies used the term ‘breakaway skills’ or ‘breakaway techniques’ to describe physical disengagement skills (46–48, 52); although two of these (47, 52) subsequently did not describe teaching skills or techniques which would usually be regarded as physical disengagement skills. Two training programmes referred to ‘self-defence manoeuvres’ (44, 46). Craig and Sanders (2018) described blocking techniques and alternative methods including the use of pillows, cushions, beanbags and other soft objects to deflect kicks, hits and slaps (45). A total of seven breakaway or ‘disengagement’ techniques were described in five studies in regards to: hair pulling (34, 48, 49); choking or strangulation (34, 48, 53); punching (49, 53, 57); wrist grabs (34, 48); biting, (34, 48); kicking (49); and headlocks (49).

### Statistical analysis

Twelve studies reported reliability data for at least their main dependent measures (30, 34, 43, 44, 47, 50–54, 56, 57). Five studies did not report reliability data for their main dependent measures (45, 46, 48, 49, 55). One study reported reliability data for staff knowledge measures (30). One study used a patient restraint written test with high inter-rater reliability, but reported no test–retest reliability measures (54). One study reported validity and reliability for a questionnaire on knowledge about physical restraint (44). Five studies used measures of staff confidence with acceptable reliability (30, 34, 47, 50, 56), while two studies did not report on reliability for this measure (48, 51).

Seven studies solely used parametric statistics (34, 43, 46, 48, 53, 54, 56), six studies used nonparametric statistics only (30, 47, 49, 50, 52, 55), and one study used a combination of parametric and non-parametric statistical analyses (51). Two studies reported descriptive statistics (45, 57). One study was unspecified ‘General Estimation Equation’ (44).

Statistical significance was reported in 15 studies (30, 34, 43, 44, 46–56). Two studies reported descriptive statistics only (45), (57): Craig and Sanders (45) was a relatively large-scale study, whereas Van den Pol et al. (57) was a small study reporting on a training intervention for 13 participants.

### Methodological quality of the studies

Due to the small number of selected articles and their variability in design quality, sample sizes and statistical analysis, it was decided not to compare these studies in terms of statistical power and rank order them in terms of quality. The MMAT (39) facilitated a degree of statistical analysis to provide further qualitative interpretation. Table 3 reports the characteristics of studies as assessed by the MMAT. Thirteen of the 17 studies included control groups to evaluate the efficacy of the training programmes (30, 44, 47–57). Two of these utilised a randomised control trial (51, 55). The remaining four studies included comparison groups for evaluation of the training programmes used (34, 43, 45, 46). The study conducted by Craig and Saunders (45) met the inclusion criteria for comparison groups as one of the settings they sampled was acquired during the course of the study, which provided a natural opportunity for comparison.

**Table 3:** Methodological qualities of studies as assessed by the mixed method appraisal tool (MMAT).

	Methodological quality criteria												Overall quality score assigned
	Screening questions		Quantitative randomized controlled trials					Quantitative non-randomized					
	S1	S2	2.1	2.5	2.3	2.4	2.5	3.1	3.2	3.3	3.4	3.5	
Allen and Tynan (30)	Yes	Yes						Yes	Yes	Yes	Yes	Yes	5
Carmel and Hunter (43)	Yes	Yes						Yes	Yes	Yes	Unclear	Yes	4
Chang et al. (33)	Yes	Yes						Yes	Yes	Yes	Yes	Yes	5
Craig and Sanders (45)	Yes	Yes						Yes	Yes	Yes	Yes	Yes	5
Godfrey et al. (46)	Yes	Yes						Yes	Yes	Yes	Yes	Yes	5
Hahn, Needham, Abserhalden et al. (47)	Yes	Yes						Yes	Yes	Unclear	Yes	Yes	4
Hurlebaus and Link (48)	Yes	Yes						Yes	Yes	Unclear	No	Yes	3
Infantino and Musingo (49)	Yes	Yes						Yes	Yes	Unclear	No	Yes	3
McDonnell, Sturmey & Oliver et al. (34)	Yes	Yes						Yes	Yes	Unclear	Yes	Yes	4
McGowan, Wynaden, Harding et al. (50)	Yes	Yes						Unclear	yes	No	no	Unclear	1
Needham, Abderhalden, Zeller et al. (51)	Yes	Yes						Yes	Yes	Unclear	Yes	Yes	4
Needham, Abderhalden, Halfens et al. (52)	Yes	Yes	Yes	Yes	Yes	Unclear	Unclear						3
Phillips and Rudestam (53)								Yes	Yes	Unclear	Unclear	Yes	3
Rice, Helzel, Varney et al. (54)	Yes	Yes						Yes	Yes	No	Unclear	Yes	3
Testad, Aasland & Aarsland et al. (55)	Yes	Yes	Yes	Yes	No	Yes	No						3
Thackrey (56)	Yes	Yes						Yes	Yes	Yes	Unclear	Yes	4
Van Den Pol, Reed & Foqua et al. (57)	Yes	Yes						Yes	Yes	Yes	No	Yes	4

Key	
Screening questions	
S1	Are there clear research questions?
S2	Do the collected data allow to address the research questions?
Quantative randomised control trials	
2.1	Is randomisation appropriately performed?
2.2	Are the groups comparable at baseline?
2.2	Are there complete outcome data?
2.2	Are outcome assessors blinded to the intervention provided?
2.2	Did the participants adhere to the assigned intervention?
Quantative non-randomised	
3.1	Are participants representative of the target population?
3.2	Are measures appropriate regarding both the outcome and intervention (or exposure)?
3.3	Are there complete outcome data?
3.4	Are the confounders accounted for in the design and analysis?
3.5	During the study period, is the intervention administered (or exposure occurred) as intended?

### Reported outcomes

Twelve outcome measures were reported across the 17 studies chosen: staff knowledge, staff confidence, staff attitudes and behaviours, use of physical restraint, use of mechanical restraint, use of seclusion, use of PRN medication, staff/service user injury, staff assault rate, staff sickness, physical skill acquisition, and service user outcomes.

Staff knowledge was reported to have improved in one study (30), using questionnaire measures. One study reported no significant increase in a knowledge-based measure post training (48). One study reported that in-service education for physical restraints enhanced relevant knowledge and techniques (44). Five studies reported improvement in staff confidence (30, 34, 50, 51, 56). Two studies reported no improvements post-training in confidence (47, 48). Two studies reported no significant effect upon staff attitudes (44, 51), or behaviours post training (44).

Three studies reported reductions in the use of physical restraint post training (30, 45, 55). One study reported significant reductions in the number of mechanical restraints, but no statistically significant reduction in the use of ‘manual holds’ (46). Two studies reported a statistically significant reduction in the use of seclusion (45, 46); one reported a reduction in seclusion across the whole service (45), and the other study reported a reduction in seclusion in one site of the service (46). One study reported a significant increase in the administration of PRN medication as the use of mechanical restraint decreased (46). No studies reported staff injuries during training courses. One study reported reductions in staff injuries post training (43). Another study reported reductions in both staff and service user injuries (46). Two studies reported reductions in rates of assault on trained staff versus untrained staff after training (49, 53), and one study reported increases in assault rates post training (54). One study reported a reduction in sickness rates relating to aggression post training (54). Four studies reported acquisition of physical interventions skills on training courses as an outcome (44, 53, 54, 57). For example, Van den Pol et al. (53) reported data using unannounced assessments of physical skills competency in the workplace.

There was limited outcome data presented that described the impacts of training in physical interventions on service-users. One study noted that training did not lead to a reduction in service-users’ challenging behaviour (34). One study reported reductions in service-user injuries post training (46). Only one study reported on qualitative outcomes for service-users (45), reporting that service-user goal mastery increased by 133%.

## Discussion

The purpose of this literature review was to examine the outcomes of staff training in physical interventions across care sectors. A systematic search of the literature was conducted following PRISMA guidelines utilising main keywords “staff training,” “physical intervention,” and “physical restraint;” 190 studies were identified in the search, but only 17 met inclusion criteria relating to publication in a peer reviewed journal and utilising a comparison group. The 17 studies included in this literature review took place in a variety of sectors that comprised of services of adults and children, including psychiatric settings, intellectual and developmental disability settings, and general hospital settings. There are several methodological criticisms of the studies presented. The findings from this literature review offer an insight into the broader structural and empirical issues in evidence-based training practices within the care sectors identified above.

The studies that were included and examined in further detail for the purpose of this literature review were of relatively poor quality. Many of the studies that were reviewed did not give appropriate detail as to how the studies were conducted or to how the training programmes were administered, which calls into question their replicability. There was also a large amount of variability between studies with regard to robustness of study design, use of validated measures, methods of statistical analysis, and the outcomes being evaluated.

Many of the included studies described use of a quasi-experimental design, the sample sizes varied from small case designs, such as that employed by Van Den Pol et al. (57), to larger audits of service data, as described by Craig and Saunders (45). Several experimental evaluations of courses had strengths. Rice et al. (54) used multiple dependent variables, reported reliability data, and used a control group and one-year follow up. There are two studies (52, 55) in this literature review that described themselves as adopting a randomised control trial (RCT). Needham et al. (52) discuss a ‘cluster RCT’ in which 3 psychiatric wards are randomly assigned to the intervention condition and compared to 3 wards that act as a wait list control. Testad et al. (55) use a similar design, in which 2 residential/nursing homes are randomly allocated to receive training, and compared to 2 homes where training is not implemented. While random allocation of ward/home to intervention or treatment condition has been implemented, in practice the designs utilised in these studies do not differ significantly from the quasi-experimental designs described in the other included studies.

Many of the studies did not clearly operationalise the knowledge and skills taught to staff in the training courses being evaluated. As such, it is difficult to compare course content across the studies. Brand names of training courses (e.g., CPI, ACT, NVCI) were often used in the articles with no clear description of these courses. Without accurate descriptions of training courses it is difficult to ascertain whether accurate replication has been achieved, and it is not clear that training programmes referred to by brand names across multiple papers are delivered in a similar manner. Few papers operationally described physical disengagement (breakaway) skills and restraint procedures in sufficient detail. Only three of the papers provide task analyses of restraint procedures (34, 36, 57) that would allow a reader to have a clearer understanding of the methods that were taught to staff. Comparing different training programmes across different settings is also a problematic issue due to the large number of variables that may impact on the delivery of training. These include the relationship with the trainer/coach, the setting of delivery, and the experience of the trainers (38).

The quality of the course content also varied across the studies in the review. Some training courses were delivered over the period of several days, while other training courses were delivered in half a day or less. Where detailed descriptions of training courses were included some studies did demonstrate strengths of course content and teaching methods. Van den Pol et al. (53) used task analyses and videotaped models of the intervention procedures used (57). In this study, behavioural skills training was used to train staff to implement restraint procedures and to train other staff, and use of unannounced observations in the workplace to observe implementation was described (57). Philips and Rudestam’s (53) describe the use of role play and rehearsal, and use of observer ratings of staff behaviour and fear. Two studies provided ongoing training and supervision for the trainees to support staff in managing crises (45, 46), with one study discussing how debriefing was utilised during these coaching periods to identify antecedents. Another study provided follow up, but the staff who had been trained were no longer employed when follow up occurred (57).

Training aims range widely between the studies and include increasing staff skill, knowledge, and confidence, reducing staff fear, reducing the use of physical interventions utilised by staff, increasing the use of other forms of interventions, reducing assault rates, reducing service user and staff injuries and associated costs, and reducing staff sick days. Each of these aims implies the use of different measures, and it is not surprising that outcomes measures in these studies vary dramatically with most utilising multiple measures. Although the MMAT questions described in Table 3 demonstrate that overall the measures were appropriately utilised statistically to determine outcomes based on the interventions, there are some difficulties with measurement. There are a number of outcome measures that should be refined and standardised. Nomothetic measures, such as rates of use of restraint, seclusion or other physical intervention, staff or service user injury or staff sick days, are influenced by definitional issues that affect how incidents are reported and recorded (11). Measures of psychological constructs, such as staff confidence, staff attitudes, or staff perception of aggressive behaviour, vary between studies as to whether they have established reliability and validity, or have been developed specifically for the purposes of the study. There is also variation in regard to reporting of reliability and validity data. This reflects that there is a need of multiple outcome measures that have good construct validity in the area of behaviour management within care settings (24, 38).

Outcome measures that focus on the impact of physical interventions on consumers of services were absent for the majority of these studies. Three studies reported on impacts on service-users (34, 45, 46), with only one of these focusing on qualitative outcomes for service-users (45). In a longitudinal service evaluation of a restraint reduction programme, Craig and Sanders (45) found that over a period of 12 years consumers’ ‘goal mastery’ increased by 133%. In recent years, there has been an increasing emphasis placed on the voices of service-users to encourage a more person-centred approach to crisis management (58, 59). Hawsawi et al. (60) reviewed literature on nurses’ and consumers’ shared experiences of seclusion and restraint. The inclusion of consumer views as a meaningful outcome would represent a significant improvement in the quality of studies that focus on staff training in physical interventions.

Another outcome variable is the impact of training in real-world settings. Staff training is influenced by a wide range of variables. Understanding the generalisation to and impact in real-world settings is critical to meaningful outcomes. The impact of training may have unforeseen consequences. A training course which results in increased acquisition of physical intervention skills and reduction in client incidents may still not be judged adequate if it also results in increases in staff and service user injuries, staff turnover, and associated costs. Similarly, a course that does not impact client incidents may still have benefits, such as reduced staff injuries associated with increased use of appropriate safe forms of restraint.

Staff training outcomes are not just determined by the delivery of a programme in a classroom setting. There are a wide range of organisational variables which will influence outcomes. At the organisational level, various models have been shown to reduce the use of physical restraints, for example multicomponent models such as ‘Six Core Strategies’ (61). Multicomponent group interventions include a range of activities such as senior leadership focus, target setting, monitoring, and reviewing outcomes data at all levels, feedback to frontline teams, in addition to staff training (22, 61). Two studies included in this review evaluate training as part of a multi-component model (44, 45). Craig and Sanders (45) report a 97% decrease in use of restraint and a 100% decrease in use of seclusion over a 12-year period of organisational change within psychiatric services, while Chang et al. (44) report an increase in staff knowledge and correct use of techniques in an ICU setting. Other studies have also found that group level interventions led to moderate to large reductions in the use of restraints (8, 61–63). Duxbury et al. (63) reported restraint reductions of 22% on mental health wards, with some wards showing a 60% reduction.

Staff training evaluations need to include an emphasis on the organisational impact of training (63). Single component models that focus on interventions other than staff training have also had some success in reducing the use of physical restraints, such as ‘SafeWards’ (64). Bowers et al. (62) reported on a cluster randomised control group trial with an average reduction of 15% of ‘conflict’ events and 26.4% reduction in ‘containment’ events following an experimental intervention focused upon staff interactions within mental health services. Lickiewicz et al. (65) implemented elements of the SafeWards programme in Poland, and reported that the number of patients restrained during the course of the study decreased by 34%.

Future research that focuses on training in physical interventions needs to address the following areas. First, training courses should be explicit in their aims and provide sufficient content to inform researchers. Second, the integrity of the independent variable needs to be acknowledged and clearly defined. Third, evaluations should use experimental designs that include adequate control groups and follow-up measures *in situ*. Fourth, studies need to use measures that are, where possible, empirically reliable and valid. Fifth, there needs to be a stronger emphasis on the implementation, generalisation, maintenance of skills after training in the workplace, and specifically how they are monitored in an organisational context. Sixth, the views of consumers as well as staff need to be strongly considered as outcome measures in future studies.

The use of randomised controlled designs is considered to be a ‘gold standard,’ however, there are significant barriers to adopting such a methodology in this field. Even if a standardised measurement protocol could be agreed, evaluating established training programmes is different from evaluating a new treatment or intervention. That only 17 out of a 190 studies identified met inclusion criteria relating to scientific rigor (and only three in last 10 years) indicates a lack of a robust evidence base for staff training in physical interventions. In practice, these training programmes are already in use without an established evidence base. In the European Union, in 2011 about 23 million persons were employed in health and social care, which made up 10.4% of the total workforce (66). Conservatively speaking, if only 1% of this workforce received either standard or refresher training annually, that would mean a minimum of 230,000 people a year receive training in physical interventions of some form. A figure of 5% (which may not be unreasonable) would produce 1 million people.

Evidence-based practice is considered to be the cornerstone of the scientist practitioner model. Consideration needs to be given to balancing the evidence-based practice for staff training in physical interventions by supplementing it with the construct of practice-based evidence (67). Practice-based evidence incorporates the complexities of real-world clinical practice by documenting and measuring outcomes as they occur (68). If we are to develop methodologies moving forward that reduce the use of physical interventions, this will require an approach where best practice in restraint reduction and elimination is evaluated through practice-based designs such as service audits and individualised case designs. This equally applies to studies which examine staff training that includes physical interventions.

Understanding practice in real world settings (as opposed to teaching/classroom settings) is central to understanding the use, application and ultimately the reduction of restrictive practices. Deveau and Leitch (21) suggested that ‘practice leadership’ and the prevailing culture within staff teams is likely to impact upon restraint and physical intervention use. There is also an opportunity to expand the methodology to include longitudinal approaches and qualitative methodologies that assist in understanding processes surrounding the use of physical interventions. There would still be a need for well-designed empirical studies that evaluate the impact of staff training in physical interventions: these should be viewed as necessary, but not sufficient to, reduce the use of such practices.

In conclusion, this review has highlighted the crude and limited nature of the research literature that focuses on staff training in physical interventions across care settings. If we are to provide clearer evidence for meaningful outcomes, then there is a need for clear and robustly designed studies. At present, the vast majority of training in physical interventions that takes place around the world could be described by researchers as in effect ‘unlicensed products.’

## Author contributions

AM was the main author and supervisor of the project. MO’S, SB-P, HM, and RD contributed to the analysis and production of the final script. All authors contributed to the article and approved the submitted version.

## Conflict of interest

The lead researcher, AM, is also a director of Studio 3 Training Systems Ltd. Authors MO’S, SB-P and HM were employed by company Studio 3 Training Systems Ltd.

The remaining author declares that the research was conducted in the absence of any commercial or financial relationships that could be construed as a potential conflict of interest.The authors declare that this study received funding from Studio 3 Training Systems Ltd. The funder had the following involvement in the study: The lead researcher, Professor Andrew McDonnell, is a director of Studio 3 Training Systems Ltd. Authors Marion O’Shea, Stephanie Bews-Pugh and Hannah McAuliffe were employees of Studio 3 Training Systems Ltd at the time of their contribution to the literature review. The remaining author, Roy Deveaux, does not have a commercial or financial relationship with the funders.

## Publisher’s note

All claims expressed in this article are solely those of the authors and do not necessarily represent those of their affiliated organizations, or those of the publisher, the editors and the reviewers. Any product that may be evaluated in this article, or claim that may be made by its manufacturer, is not guaranteed or endorsed by the publisher.
